# Rabies Virus Infection Induces Microtubule Depolymerization to Facilitate Viral RNA Synthesis by Upregulating HDAC6

**DOI:** 10.3389/fcimb.2017.00146

**Published:** 2017-04-26

**Authors:** Jie Zan, Song Liu, Dong-Nan Sun, Kai-Kun Mo, Yan Yan, Juan Liu, Bo-Li Hu, Jin-Yan Gu, Min Liao, Ji-Yong Zhou

**Affiliations:** ^1^Key Laboratory of Animal Virology of Ministry of Agriculture, Zhejiang UniversityHangzhou, China; ^2^Institute of Immunology, Nanjing Agricultural UniversityNanjing, China; ^3^Collaborative Innovation Center and State Key Laboratory for Diagnosis and Treatment of Infectious Diseases, The First Affiliated Hospital, Zhejiang UniversityHangzhou, China

**Keywords:** rabies virus, matrix protein, microtubule depolymerization, viral RNA synthesis, acetylated modification

## Abstract

Rabies virus (RABV) is the cause of rabies, and is associated with severe neurological symptoms, high mortality rate, and a serious threat to human health. Although cellular tubulin has recently been identified to be incorporated into RABV particles, the effects of RABV infection on the microtubule cytoskeleton remain poorly understood. In this study, we show that RABV infection induces microtubule depolymerization as observed by confocal microscopy, which is closely associated with the formation of the filamentous network of the RABV M protein. Depolymerization of microtubules significantly increases viral RNA synthesis, while the polymerization of microtubules notably inhibits viral RNA synthesis and prevents the viral M protein from inducing the formation of the filamentous network. Furthermore, the histone deacetylase 6 (HDAC6) expression level progressively increases during RABV infection, and the inhibition of HDAC6 deacetylase activity significantly decreases viral RNA synthesis. In addition, the expression of viral M protein alone was found to significantly upregulate HDAC6 expression, leading to a substantial reduction in its substrate, acetylated α-tubulin, eventually resulting in microtubule depolymerization. These results demonstrate that HDAC6 plays a positive role in viral transcription and replication by inducing microtubule depolymerization during RABV infection.

## Introduction

Rabies virus (RABV) is a prototypical member of the *Lyssavirus* genus of the *Rhabdoviridae* family, and the causative agent of rabies. Rabies is associated with severe neurological symptoms and high mortality rate, causing more than 50,000 human deaths annually, mainly in Asia and Africa (Sudarshan et al., 2007). Although rabies has been studied for over 100 years, it remains incurable and a serious threat to human health. To develop effective antiviral drugs, a deeper understanding of the pathogenic mechanisms of RABV infection is required.

The life cycle of RABV occurs exclusively in the cytoplasm with the transcription of five viral genes that encode the viral proteins: nucleoprotein (N), phosphoprotein (P), matrix protein (M), glycoprotein (G), and the viral RNA polymerase (L), respectively. The N, P, and L proteins, together with the viral RNA genome, form a helical ribonucleoprotein (RNP) complex that is responsible for viral RNA transcription and replication (Albertini et al., 2011). The M protein recruits RNPs to the cell membranes and interacts with the G protein to facilitate virion budding and release (Mebatsion et al., 1999). Furthermore, the M protein has been reported to regulate the balance of viral RNA synthesis and be related to the pathogenicity of RABV (Finke and Conzelmann, 2003; Pulmanausahakul et al., 2008; Wirblich et al., 2008).

The degeneration of neuronal processes is considered to play an essential role in the fatal neurological symptoms of rabies (Baloul and Lafon, 2003; Scott et al., 2008; Kojima et al., 2009). An increasing number of studies indicate that microtubules are vital to the development and maintenance of axons and dendrites throughout the life of the neuron, and microtubule abnormalities are closely associated with neurodegenerative diseases (Matamoros and Baas, 2016; Stevenson et al., 2016). Thus, we hypothesized that the neuronal degeneration that occurs during RABV infection might be linked to microtubule abnormalities.

Microtubules are hollow, cylindrical structures composed of associated protofilaments of α and β-tubulin dimers and play important roles in multiple cellular processes, such as intracellular transport, signaling pathways, and cellular division (Downing, 2000; Hammond et al., 2008; Akhmanova and Steinmetz, 2015). The functional versatility of microtubules is dependent on microtubule dynamics, which are regulated by microtubule-associated proteins (MAPs; Akhmanova and Steinmetz, 2015; Alfaro-Aco and Petry, 2015). Among these, MAP4 binds to the microtubule lattice to stabilize microtubules, and thereby prevent depolymerization (Kadavath et al., 2015). Alternatively, stathmin depolymerizes microtubules by sequestering tubulin subunits and promoting microtubules shrinkage, so as that the growth of microtubules was blocked. This procedure was termed as “microtubule catastrophe” (Howell et al., 1999). In addition, microtubule dynamics have been found to be regulated by post-translational modifications, such as acetylation, phosphorylation, or palmitoylation of α-tubulin (Hammond et al., 2008; Song and Brady, 2015). Among these modifications, the acetylation which occurs on lysine 40 of α-tubulin is fairly common and considered to be a well-known marker of stable microtubules (L'Hernault and Rosenbaum, 1985; Perdiz et al., 2011). Furthermore, α-tubulin can be deacetylated by histone deacetylase 6 (HDAC6), a primary α-tubulin deacetylase (Matsuyama et al., 2002; Yang et al., 2013).

Numerous viruses have been reported to utilize microtubules to facilitate the intracellular transport of virions or subviral particles, including human immunodeficiency virus type 1 (HIV-1), vaccinia virus, herpesvirus, and circovirus (Sanderson et al., 2000; Nishi et al., 2009; Pasdeloup et al., 2013; Cao et al., 2015; Fernandez et al., 2015). In addition, several viruses have been reported to take advantage of microtubules to regulate the formation of viral inclusion bodies, such as reovirus and orthopoxvirus (Parker et al., 2002; Howard and Moss, 2012). Conversely, some viruses induce microtubule depolymerization to facilitate viral infection, [e.g., rotavirus, Epstein-Barr virus, and Sendai virus (SeV)] (Ogino et al., 2003; Martin et al., 2010; Liu et al., 2013). Although a recent study demonstrates that α-tubulin is incorporated into RABV particles (Tu et al., 2015), the role of the microtubule cytoskeleton in the process of RABV infection, especially regarding viral transcription and replication remains poorly understood. Therefore, the aim of this study was to investigate: (1) the effects of RABV infection on the microtubule cytoskeleton; and (2) the role of microtubule dynamics in viral transcription and replication.

## Materials and methods

### Cells, virus, and reagents

Mouse neuroblastoma N2a cells, baby hamster kidney (BHK-21) cells, and human embryonic kidney epithelial (HEK) 293T cells were cultured in DMEM supplemented with 10% fetal bovine serum (Gibco/Invitrogen, USA) at 37°C with 5% CO_2_. The challenge virus standard-11 strain of fixed rabies virus (CVS) was propagated in N2a cells. All the cells and virus used in this study were stored in our laboratory. Cells were infected with CVS at a multiplicity of infection (MOI) of 1 and the supernatant was harvested after 72 h post-infection (hpi). Virus preparations were titrated on N2a cells, and then stored at −80°C.

### Drug treatment

The chemicals used to treat the RABV-infected N2a cells included the microtubule-depolymerizing drug nocodazole (Noco) (S1765; Beyotime, China), the microtubule-polymerizing drug paclitaxel (Taxol) (S1150; Selleckchem, USA), the histone deacetylase (HDAC) inhibitor trichostatin A (TSA) (T1952; Sigma) and the HDAC inhibitor sodium butyrate (NaBut) (S1539; Beyotime). The N2a cells were infected with RABV for 4 h and then treated with the various drugs. The solvent dimethyl sulfoxide (DMSO) was used as a control.

### Cell viability assay

N2a cells cultured in 96-well-plates were incubated with each drug or DMSO for 20 h. Cell viability was measured using a CCK-8 Cell Counting Kit (A311-02; Vazyme, China) and expressed as the percent of the control culture as described previously (Zan et al., 2016a).

### Transient transfection

N2a cells were seeded into 6-well-plates or 35 mm glass bottom dishes (Shengyou Biotechnology Co. Ltd., Hangzhou, China). On the following day, the N2a cells were transfected with p-CMV-Myc-M (Zan et al., 2016b) or the pCMV-myc empty vector using Exfect Transfection Reagent (Vazyme Biotech Co. Ltd., Nanjing, China), and the transfection procedure was carried out according to the manufacturer's instructions.

### Confocal microscopy

N2a, BHK-21, or 293T cells grown overnight into 35-mm glass bottom dishes were infected with RABV (MOI = 1) or not. At the indicated hours post-infection (hpi), the cells were fixed with 4% paraformaldehyde, permeabilized with 0.5% Triton X-100 in PBS, and incubated at 4°C with primary antibodies overnight. Cells were then incubated with FITC-conjugated-secondary antibodies (KPL) and/or Alexa Fluor 546-conjugated secondary antibodies (Invitrogen) at 37°C for 1 h. Cellular nuclei were stained with 10 μg/ml DAPI (Roche) for 5 min, and viewed by an LSM780 laser scanning confocal microscopy (Carl Zeiss). All images were taken using multitrack scanning for each fluorophore to prevent bleed-through. FITC fluorescence was detected after excitation at 488 nm with an emission long-band filter at 505–530 nm (green). Alexa Fluor 546 fluorescence was detected after excitation at 561 nm with an emission long-pass filter at 550–600 nm (red). DAPI was detected after excitation at 405 nm with an emission long-pass filter at 445–450 nm (blue). The pinholes were set to an Airy unit of 1 (equal in size to an Airy disk). Images were acquired with the identical capture settings and analyzed using Zen 2012 software (Zeiss). Rabbit anti-α-tubulin antibody (11224-1-AP; Proteintech, China), rabbit anti-acetylated α-tubulin antibody (5335S, Cell Signaling Technology), mouse anti-RABV-N antibody, and mouse anti-RABV-M antibody (Zan et al., 2016a) were used as primary antibodies.

### RNA preparation and quantitative real-time PCR

N2a cells were infected with RABV (MOI = 1) for 4 h and then treated with either the drugs or DMSO for another 20 h. The total RNA was extracted from the cells using an RNA isolater Total RNA Extraction Reagent (R401-01, Vazyme). RNA (500 ng) from each sample was reverse-transcribed into cDNA using a PrimeScript® RT reagent kit (Takara). Quantitative real-time PCR was performed on the 7500 real-time PCR system (Applied Biosystems) using AceQ qPCR SYBR Green Master Mix (Q111-02, Vazyme). qPCR primers were used as described previously (Zhang et al., 2013; Xu et al., 2016).

### Determination of virus titer

N2a cells were infected with RABV (MOI = 1) for 4 h and then treated with either the drugs or DMSO for another 20 h. The viral titers from culture supernatants were determined by viewing the infected cells under a fluorescent microscopy and calculating the TCID_50_ per 0.1 mL as described previously (Zan et al., 2016a).

### Western blotting

Western blotting was performed as previously described (Zhang et al., 2013). Briefly, cells were lysed with an NP40 lysis buffer (P0013F, Beyotime) after infection or transfection for the indicated times. Lysates were collected, and the protein concentrations were determined with BSA protein assay kit (P0010S, Beyotime). Equivalent amounts of cell lysates (80 μg) were resuspended in 5 × SDS-PAGE loading buffer (P0015, Beyotime) and boiled for 10 min. After centrifugation, the soluble cell lysates were separated on 12% SDS-PAGE gels, electro-transferred onto 0.22-μm-pore-size nitrocellulose membranes (GE Healthcare), and subjected to immunoblot analysis. Membranes were blocked in PBS containing 0.05% Tween 20 (PBST) and 5% skimmed milk for 1 h. Membranes were then incubated with primary antibody overnight at 4°C, washed three times with PBST for 5 min each time, and incubated with an appropriate secondary antibody conjugated to horseradish peroxidase (HRP) (KPL) for 1 h at 37°C. Finally, membranes were washed three more times with PBST before visualization was performed using SuperSignal West Pico chemiluminescent substrate (34079, Thermo) under the conditions recommended by the manufacturer. Images were captured using optimal auto-exposure settings on a chemiluminescent imaging system (Cell Biosciences, USA) and the densities of protein bands were normalized against the GAPDH signal and quantified using ImageJ software (National Institutes of Health, USA). Rabbit anti-HDAC6 antibody (ab1440, Abcam), rabbit anti-acetylated α-tubulin antibody (5335S, Cell Signaling Technology), rabbit anti-α-tubulin antibody (11224-1-AP, Proteintech), mouse anti-RABV-N antibody, rabbit anti-Myc antibody (0912-2, HuaAn Biotechnology, China), rabbit anti-stathmin antibody (11157-1-AP, Proteintech), rabbit anti-MAP4 antibody (11229-1-AP, Proteintech), and anti-glyceraldehyde-3-phosphate dehydrogenase (GAPDH) antibody (R1210-1, HuaAn Biotechnology, China) were used as the primary antibodies.

### Statistical analysis

The data were statistically analyzed and graphed using GraphPad Prism 5 (GraphPad Software, San Diego, CA). All of the results are presented as the means ± standard deviations. Statistically, significant differences between groups were determined by a Student's *t*-test. ^*^P < 0.05 and ^**^P < 0.01.

## Results

### RABV infection damages the host microtubule cytoskeleton

**Figure 1:**
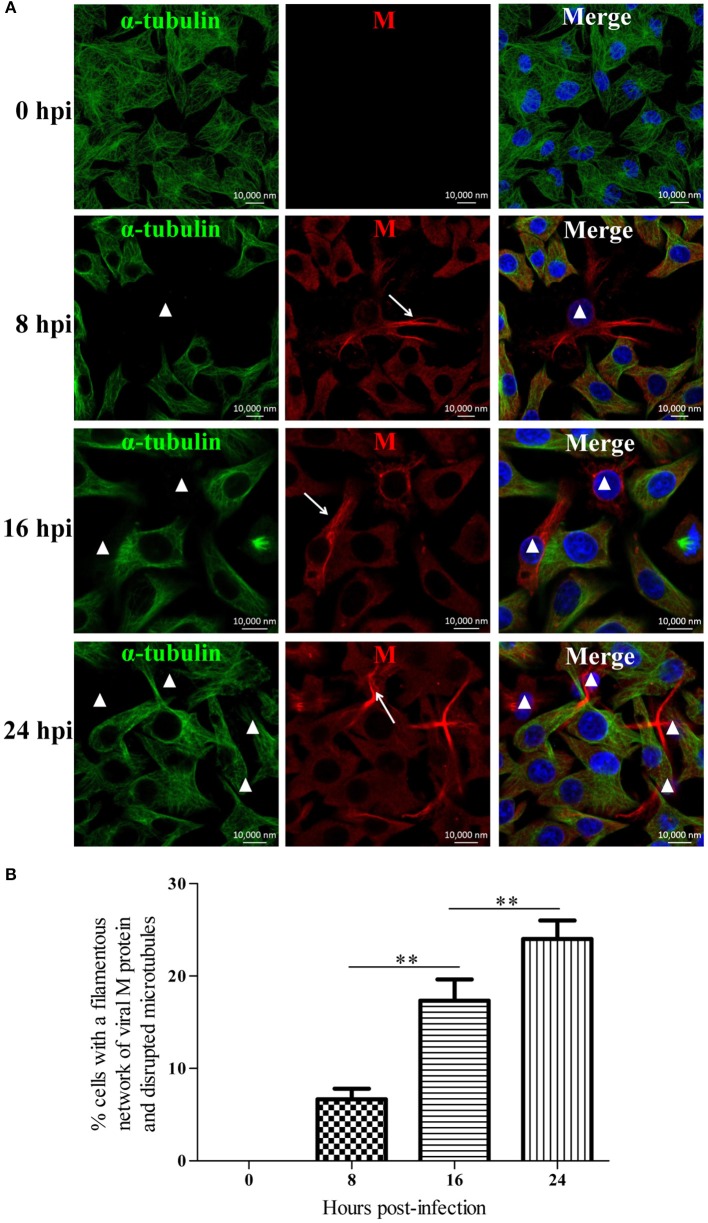
**RABV infection damages the host microtubule cytoskeleton. (A)** N2a cells were infected by RABV at an MOI of 1 for 0, 8, 16, or 24 h. Subsequently, the cells were fixed, permeabilized, and incubated with the anti-α-tubulin antibody (green) and anti-RABV-M antibody (red). The nuclei (Nuc) were stained with DAPI (blue). The cells were analyzed using a laser scanning confocal microscopy. The white arrows represent the filamentous network of viral M protein in RABV-infected cells and the white triangles indicate the cells which have a filamentous network viral M protein and disrupted microtubules. Scale bars, 10,000 nm. **(B)** Three random microscopic fields having at least 50 cells each were chosen to calculate the number of cells with a filamentous network of viral M protein and disrupted microtubules at the indicated time points. Results were expressed as percentage of viral filamentous network-positive and cellular microtubules-negative cells relative to DAPI-positive cells. Data are represented as the means ± SD (*n* = 3; ^**^represents *P* < 0.01).

To investigate the effects of RABV infection on the host microtubule cytoskeleton, we performed an immunofluorescence analysis of RABV-infected N2a cells at 0, 8, 16, and 24 hpi with antibodies against the viral M protein and α-tubulin (Figure 1A). In the mock-infected cells at 0 hpi, we observed an intense green fluorescence throughout the cytoplasm, reflecting a high concentration of microtubules (Figure 1A). In the RABV-infected cells at 8 hpi, the viral M protein had diffused throughout the cytoplasm, and in some of the infected cells, M protein was clustered to form an intensely filamentous structure which appeared predominantly as a network in the cytoplasm, called “filamentous network” (indicated by the white arrows in Figure 1A and Supplementary Figure 1). This result was consistent with our previous studies (Zan et al., 2016a,b). Surprisingly, the microtubules were virtually undetectable in the cells in which the viral M protein formed a filamentous network (indicated by the white triangles, Figure 1A). This phenomenon was also observed in RABV-infected cells at 16 and 24 hpi, and the number of cells with a filamentous network of viral M protein and disrupted microtubule at 8, 16, and 24 hpi was about 7, 17, and 24%, respectively, which was progressively increased in a time-dependent manner (Figures 1A,B). This suggests that the disruption of the microtubule cytoskeleton induced by RABV infection is likely associated with the formation of the M protein filamentous network. Moreover, similar results were also obtained with the BHK-21 and 293T cells (Supplementary Figure 2). These results indicate that RABV infection damages the host microtubule cytoskeleton and is associated with the accumulation of the viral M protein.

### Depolymerization and polymerization of microtubules have an inverse effect on the RNA synthesis of RABV

**Figure 2:**
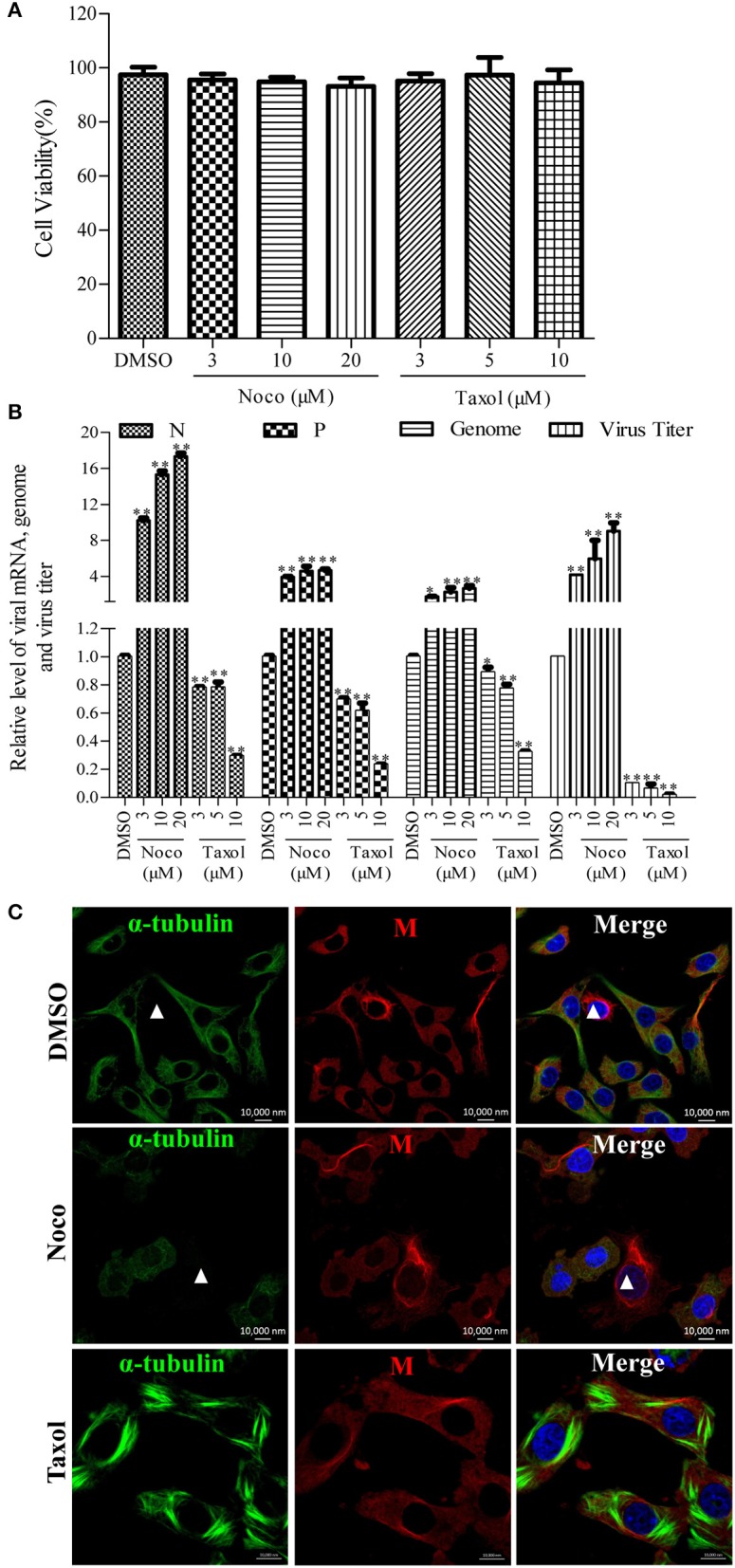
**Effects of Noco or Taxol treatment on viral RNA synthesis. (A)** Detection of the cellular viability of N2a cells incubated with Noco, Taxol, or DMSO (as control) for 20 h by CCK-8. **(B)** The quantitative analysis of viral N and P mRNA, viral genomic RNA, and viral titer. The N2a cells were infected with RABV for 4 h and then incubated with Noco, Taxol, or DMSO for another 20 h. The cells were collected for viral RNA extraction. Quantitative real-time PCR was performed to detect the viral genes and genome. The titers of infectious RABV progeny from culture supernatants were determined by TCID_50_. **(C)** The distribution of the viral M protein (red) and microtubule cytoskeleton (green) in RABV-infected N2a cells in the presence of Noco, Taxol, or DMSO at 24 hpi. Nuclei (Nuc) were stained with DAPI (blue). The white triangles indicate the cells which have a filamentous network viral M protein and disrupted microtubules. Scale bars, 10,000 nm. Data are represented as the means ± SD (*n* = 3; ^*^represents *P* < 0.05, ^**^represents *P* < 0.01).

Since we found that RABV infection damages the microtubule cytoskeleton (Figure 1), we next wanted to elucidate the effects of microtubule dynamics on the transcription and replication of RABV. For this purpose, N2a cells were infected with RABV for 4 h to allow sufficient time for viral entry into the cytoplasm and intracellular transport (Xu et al., 2015). Subsequently, the cells were then treated with the microtubule-depolymerizing drug Noco, the microtubule-polymerizing drug Taxol, or DMSO for another 20 h, respectively. As shown in Figure 2A, treatment with Noco or Taxol did not substantially affect cellular viability. Surprisingly, the levels of the viral N and P mRNA, viral genomic RNA, and infectious RABV progeny were all significantly increased in Noco-treated cells, but were notably reduced in Taxol-treated cells (Figure 2B). These findings suggest that RABV-induced microtubule depolymerization promotes viral RNA synthesis.

Furthermore, we investigated whether microtubule dynamics affect the filamentous network formation of the viral M protein. As observed in Figure 2C, microtubule depolymerization neither inhibited the filamentous network formation of the viral M protein nor blocked the RABV-induced disruption of the microtubule cytoskeleton in Noco-treated cells (indicated by a white triangle). However, the filamentous network formation of the viral M protein was significantly inhibited by small amounts of M protein clustered only near the nucleus in Taxol-treated cells, where the microtubules polymerized into bundles, compared to that in DMSO-treated cells (Figure 2C). Thus, we hypothesized that RABV infection induces microtubule depolymerization. In addition, we also found that microtubule depolymerization or polymerization did not affect the formation of Negri bodies (NBs; Supplementary Figure 3; the sites of RABV transcription and replication) which accumulated by the viral N protein in the cytoplasm, and was consistent with a previous study (Lahaye et al., 2009). Taken together, these results indicate that microtubule depolymerization promotes the RNA synthesis of RABV, but does not affect the formation of the viral M protein filamentous network.

### RABV infection reduces the acetylated modification of microtubules through the upregulation of HDAC6

**Figure 3:**
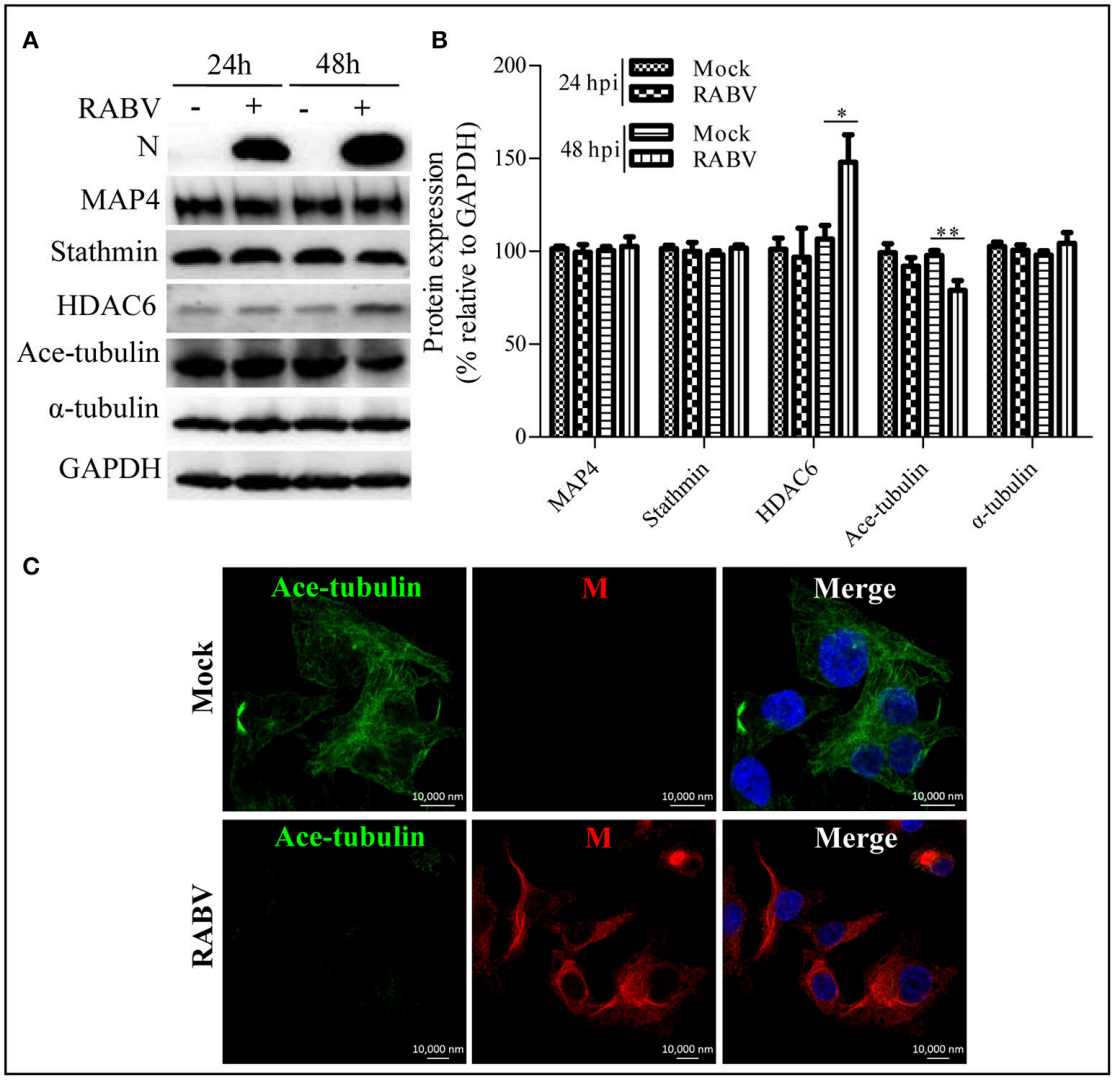
**Effects of an RABV infection on the acetylated modification of microtubules. (A)** N2a cells were infected with RABV or not. At 24 and 48 hpi, the cells were lysed and subjected to Western blotting to determine the expression of the viral N protein, MAP4, stathmin, HDAC6, ace-tubulin, α-tubulin, and GAPDH (as loading control). **(B)** The relative expression levels of MAP4, stathmin, HDAC6, ace-tubulin, and α-tubulin were calculated by normalizing to that of GAPDH, respectively. **(C)** The distribution of the viral M protein (red) and ace-tubulin (green) in RABV-infected or mock-infected N2a cells at 24 hpi. The cell nuclei (Nuc) stained with DAPI (blue). Scale bars, 10,000 nm. The data are presented as the means ± SD (*n* = 3; ^*^represents *P* < 0.05, ^**^represents *P* < 0.01).

Microtubule dynamics are regulated by MAPs (e.g., MAP4 and stathmin) and are closely associated with post-translational modifications; in particular, α-tubulin acetylation is recognized as a well-known maker of stable microtubules and is governed by HDAC6 (Howell et al., 1999; Matsuyama et al., 2002; Perdiz et al., 2011; Yang et al., 2013; Akhmanova and Steinmetz, 2015; Alfaro-Aco and Petry, 2015; Kadavath et al., 2015). To clarify RABV-induced microtubule depolymerization, we next examined the expression levels of MAP4, stathmin, HDAC6 and its substrate, acetylated α-tubulin (ace-tubulin) in RABV-infected or mock-infected N2a cells at 24 and 48 hpi. The results in Figures 3A,B revealed that the expression levels of MAP4 and stathmin remain relatively stable, while the HDAC6 expression levels significantly upregulated at 48 hpi. Moreover, there was a decrease in the level of ace-tubulin expression in RABV-infected cells, compared to those in the mock-infected cells. In addition, the acetylated microtubules were weakly stained or nearly undetectable in the RABV-infected cells but not in the mock-infected cells as indicated by confocal microscopy (Figure 3C). Collectively, these results indicate that RABV infection induces upregulation of HDAC6 expression to reduce ace-tubulin expression, and results in destabilizing the microtubule cytoskeleton.

### Inhibition of the deacetylase activity of HDAC6 decreases RABV RNA synthesis

**Figure 4:**
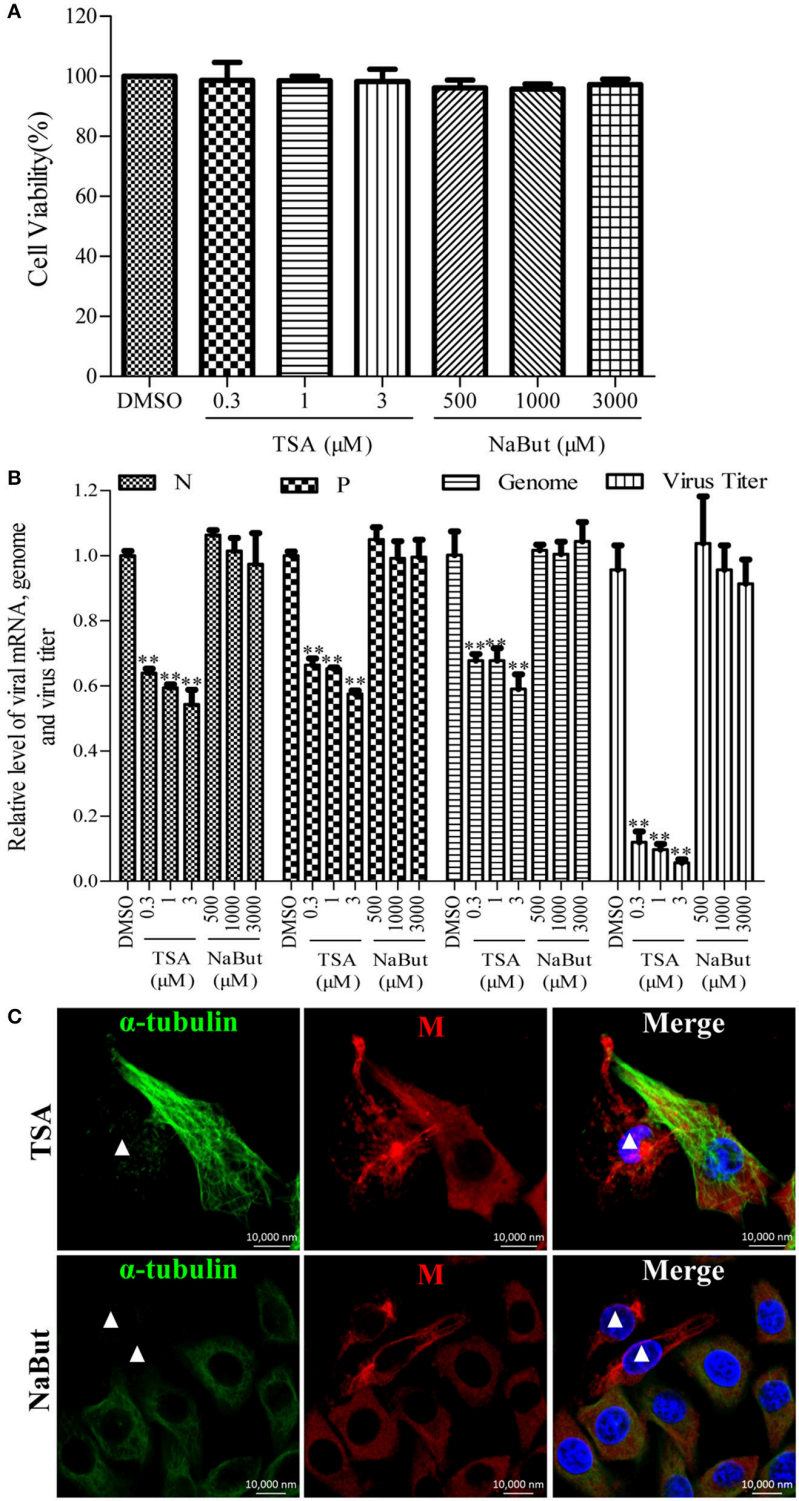
**Effects of inhibiting the deacetylase activity of HDAC6 on viral RNA synthesis. (A)** The detection of the cell viability of N2a cells incubated with TSA, NaBut, or DMSO (as control) for 20 h by CCK-8. **(B)** N2a cells were infected with RABV for 4 h and then incubated with TSA, NaBut, or DMSO for another 20 h and the cells were collected for viral RNA extraction. Quantitative real-time PCR was performed to detect the viral genes and genome. The titers of infectious RABV progeny from the culture supernatants were determined by TCID_50_. **(C)** The distribution of the viral M protein (red) and microtubule cytoskeleton (green) in RABV-infected N2a cells in the presence of TSA or NaBut at 24 hpi. Nuclei (Nuc) were stained with DAPI (blue). The white triangles indicate the cells which have a filamentous network viral M protein and disrupted microtubules. Scale bars, 10,000 nm. Data are represented as the means ± SD (*n* = 3; ^**^represents *P* < 0.01).

To investigate whether the deacetylase activity of HDAC6 has any effect on the RNA synthesis of RABV, the N2a cells were infected with RABV for 4 h and then treated with TSA, NaBut, or DMSO for another 20 h. The Western blotting analysis and immunofluorescence staining (Supplementary Figures 4A,B) revealed that the ace-tubulin expression levels were significantly increased in the TSA-treated cells but not in the NaBut or DMSO-treated cells, suggesting that the inhibition of HDAC6 activity by TSA is effective. In addition, the viability of the cells treated with TSA or NaBut were not significantly affected, and were comparable to that of the DMSO-treated cells (Figure 4A). As expected, treatment with TSA significantly inhibited viral RNA synthesis and downregulated the viral titer compared to treatment with NaBut or DMSO (Figure 4B). This suggests that the deacetylase activity of HDAC6 is important for viral RNA synthesis during RABV infection.

Furthermore, we investigated whether the inhibition of the HDAC6 deacetylase activity affected the filamentous network formation of the viral M protein. As observed in Figure 4C, treatment with TSA neither inhibited the filamentous network formation of the viral M protein nor blocked the RABV-induced disruption of the microtubule cytoskeleton (indicated by a white triangle), suggesting that HDAC6 plays a partial, but not a complete, role in RABV-induced microtubule depolymerization. In addition, we also observed that the inhibition of HDAC6 deacetylase activity did not affect the formation of NBs (Supplementary Figure 3). Taken together, these results indicate that the inhibition of HDAC6 deacetylase activity decreases the RNA synthesis of RABV.

### M protein alone induces microtubule depolymerization

**Figure 5:**
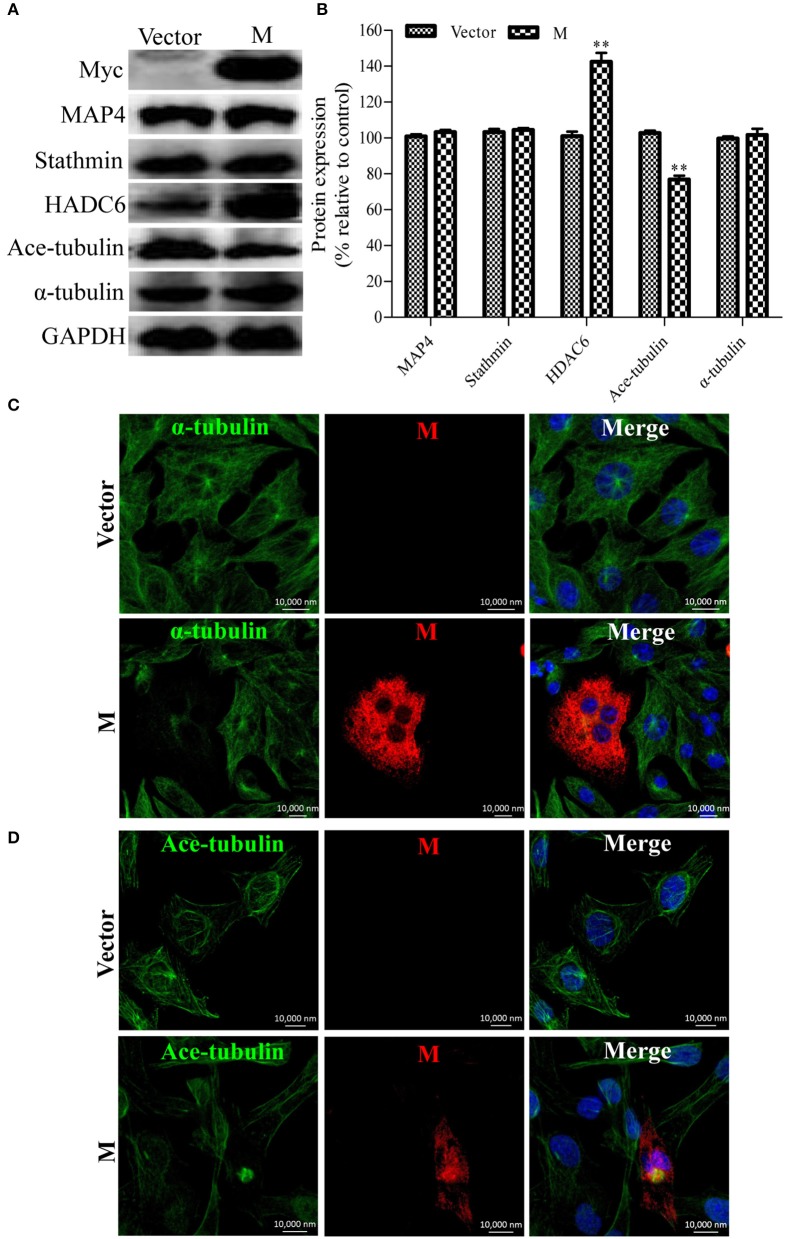
**Expression of the viral M protein alone induces microtubule depolymerization. (A)** N2a cells were transfected with p-CMV-Myc-M or the pCMV-myc empty vector (control) for 36 h, and then subjected to Western blotting to determine the expression of MAP4, Stathmin, HDAC6, ace-tubulin, α-tubulin, and GAPDH (loading control). **(B)** The relative expression levels of MAP4, stathmin, HDAC6, ace-tubulin, α-tubulin were calculated by normalizing to that of GAPDH. **(C,D)** The distribution of the viral M protein (red), microtubule cytoskeleton (green), or acetylated microtubules (green) in N2a cells transfected with p-CMV-Myc-M or the pCMV-myc empty vector. Nuclei (Nuc) were stained with DAPI (blue). Scale bars, 10,000 nm. Data are represented as the means ± SD (*n* = 3; ^**^represents *P* < 0.01).

Since the disruption of the microtubule cytoskeleton by RABV infection was closely associated with the filamentous network formation of the viral M protein (Figure 1), it was hypothesized that the M protein might be involved in the induction of microtubule depolymerization. To explore this possibility, N2a cells were transfected with p-CMV-Myc-M or the pCMV-myc empty vector (as a control) for 36 h, and then analyzed by Western blotting. As shown in Figures 5A,B, the viral M protein alone did not modify the expression of MAP4 and stathmin, but significantly upregulated the expression of HDAC6, resulting in the downregulation of ace-tubulin expression. Furthermore, we analyzed the effects of the M protein alone on the integrity of the microtubule cytoskeleton via confocal microscopy. As shown in Figures 5C,D, both the microtubules and acetylated microtubules were depolymerized with weak staining in cells expressing the viral M protein, while microtubules or acetylated microtubules remained intact with bright staining in the non-transfected or empty vector-transfected cells. Taken together, these results indicate that the M protein alone depolymerizes microtubules, and is associated with the upregulation of HDAC6 and downregulation of ace-tubulin expression.

## Discussion

The microtubule cytoskeleton plays an important role for multiple cellular functions, including cellular division, signaling, and intracellular trafficking (Downing, 2000; Hammond et al., 2008; Akhmanova and Steinmetz, 2015). Currently, numerous viruses have been reported to utilize the microtubule cytoskeleton for intracellular trafficking, including HIV-1, vaccinia virus, herpesvirus, circovirus, and RABV (Sanderson et al., 2000; Nishi et al., 2009; Pasdeloup et al., 2013; Cao et al., 2015; Fernandez et al., 2015; Xu et al., 2015). A recent study reported that α-tubulin was incorporated into RABV particles (Tu et al., 2015), suggesting that with the exception of intracellular trafficking, the microtubule cytoskeleton might play other roles during RABV infection. In the present study, we were the first to demonstrate that microtubule depolymerization induced by RABV infection facilitates viral RNA synthesis.

The rhabdoviral M protein is a multifunctional protein which plays important roles in regulating the balance between viral transcription and replication, and is associated with cellular pathogenesis, virion assembly, and budding (Mebatsion et al., 1999; Finke and Conzelmann, 2003; Pulmanausahakul et al., 2008; Wirblich et al., 2008). In addition, the M protein of vesicular stomatitis virus (VSV), another member of the *Rhabdoviridae* family, has been reported to self-assemble to form long fibers *in vitro* via the association between the N-terminal portion of the M protein and the globular domain of an adjacent M molecule (McCreedy et al., 1990; Gaudin et al., 1995, 1997; Gaudier et al., 2001). Based on similar 3-dimensional crystal structures of the M proteins from VSV and the Lagos bat virus, the highly conservative sequence of the interacting N-terminal region, as well as the globular domain of the M proteins of the *Lyssavirus* genus (Gaudier et al., 2002; Assenberg et al., 2008; Graham et al., 2008), rhabdoviral M proteins likely share the ability to self-assemble into homo-oligomers. In this study, the viral M protein of RABV was found to accumulate to form a filamentous network (Figure 1) which may indicate the display pattern of the RABV M protein self-assembly. In addition to self-assembly, the N-terminal domain of the VSV M protein has been reported to interact with tubulin, resulting in a disruption of the microtubule cytoskeleton (Melki et al., 1994). Strikingly, we observed that microtubules were nearly undetectable in RABV-infected cells where viral M protein accumulates to form the filamentous network (Figure 1). Thus, we hypothesize that the disruption of the microtubule cytoskeleton by RABV may be due to the possible interaction of the viral M protein with tubulin or the stretching forces from the filamentous network of viral M protein that physically impair the microtubule cytoskeleton. Moreover, the polymerization of microtubules substantially inhibits the assembly of the viral M protein (Figure 2C), which further confirms our above hypothesis. Although the viral M proteins of VSV and chandipura virus have been shown to interact with cellular tubulin (Melki et al., 1994; Rajasekharan et al., 2015), regrettably, we could not detect the interaction between tubulin and the RABV M protein via a co-immunoprecipitation assay (data not shown). In addition, we also demonstrated that the expression of the viral M protein alone disrupts microtubules (Figure 5), suggesting that neuronal degeneration induced by RABV infection is likely related to the disruption of microtubules.

HDAC6 is a predominant deacetylase of microtubules and has been reported to play critical roles in viral infection (L'Hernault and Rosenbaum, 1985; Matsuyama et al., 2002; Perdiz et al., 2011; Yang et al., 2013). Some viruses induce significant acetylation of α-tubulin to stabilize the microtubule cytoskeleton for their intracellular transport by regulating HDAC6, including circovirus, herpes simplex°virus, and influenza A virus (Husain and Harrod, 2011; Zhong et al., 2014; Cao et al., 2015). Recently, HDAC6 has also been reported to be involved in the replication of certain viruses. HDAC6 promotes hepatitis C virus replication and reactivates the lytic phase of HIV-1 and Kaposi's sarcoma-associated herpesvirus replication (Banerjee et al., 2012; Shin et al., 2014; Kozlov et al., 2015). Similarly, we found that HDAC6 affects RABV RNA synthesis. Moreover, the inhibition of HDAC6 by TSA significantly suppresses both RABV transcription and replication (Figure 4B). Unexpectedly, the inhibition of HDAC6 neither prevents RABV from disrupting microtubules nor blocks the formation of the filamentous network of viral M protein (Figure 4C). This finding suggests that HDAC6 may play a partial role in the RABV-induced disruption of microtubules and other unknown mechanisms for impairment of microtubules should exist during RABV infection. Although it remains unknown how RABV regulates HDAC6, the finding that HDAC6 is involved in RABV RNA synthesis is highly important.

Cellular tubulin has been reported to stimulate the viral RNA synthesis of some other negative-stranded RNA viruses, including SeV, measles virus, and VSV (Moyer et al., 1986, 1990; Ogino et al., 2003). As a negative-stranded RNA virus, RABV RNA synthesis is also mediated by tubulin. In this study, the depolymerization of microtubules significantly upregulated the transcription and replication levels of RABV (Figure 2B), while the polymerization of microtubules or the blockade of HDAC6 deacetylase activity notably inhibited viral RNA synthesis (Figures 2B, 4B). These findings suggest that RABV infection induces microtubule depolymerization into tubulin monomers, which stimulate viral RNA synthesis. Increasing studies have demonstrated that the RABV M protein is a negative regulator of transcription by condensing and recruiting viral RNP to the budding sites on the cell membrane, resulting in the inhibition of both viral transcription and replication (Finke and Conzelmann, 2003; Finke et al., 2003; Jayakar et al., 2004; Banerjee, 1987). Therefore, RABV-induced microtubule depolymerization for the stimulation of viral RNA synthesis is most likely due to the fact that tubulin dissociates the viral M protein from RNP, leading to the release of M protein inhibition on the viral RNP.

In conclusion, our study reports for the first time, that RABV infection induces microtubule depolymerization to facilitate viral RNA synthesis, and that HDAC6 is involved in this process. Although additional studies remain to be explored to reveal the molecular basis for the activation of viral RNA synthesis by microtubule depolymerization, the results of the present study deepen our understanding of RABV pathogenicity and provide novel insight into the relationship between RABV and the microtubule cytoskeleton.

## Author contributions

Design and supervise the study: JYZ, ML, JYG, and JZ. Perform the experiments: JZ, KKM, SL, DNS, YY, JL, and BLH. Prepare the manuscript: JZ, ML, JYG, and JYZ.

### Conflict of interest statement

The authors declare that the research was conducted in the absence of any commercial or financial relationships that could be construed as a potential conflict of interest.

## Abbreviations

RABV: rabies virus
RNP: ribonucleoprotein
MAPs: microtubule-associated proteins
HDAC6: histone deacetylase 6
M protein: matrix protein
Noco: nocodazole
Taxol: paclitaxel
TSA: trichostatin A
NaBut: sodium butyrate
DMSO: dimethyl sulfoxide
CVS: the challenge virus standard-11 strain of fixed rabies virus
MOI: multiplicity of infection
Ace-tubulin: acetylated α-tubulin
GAPDH: glyceraldehyde-3-phosphate dehydrogenase.
