# Supplementary material for: Rabies Virus Infection Induces Microtubule Depolymerization to Facilitate Viral RNA Synthesis by Upregulating HDAC6
Source: Front Cell Infect Microbiol. 2017 Apr 26;7:146. doi: 10.3389/fcimb.2017.00146 (PMC5405082; doi:10.3389/fcimb.2017.00146)
Supplement: Supplementary file 1 [file Presentation1.PDF]

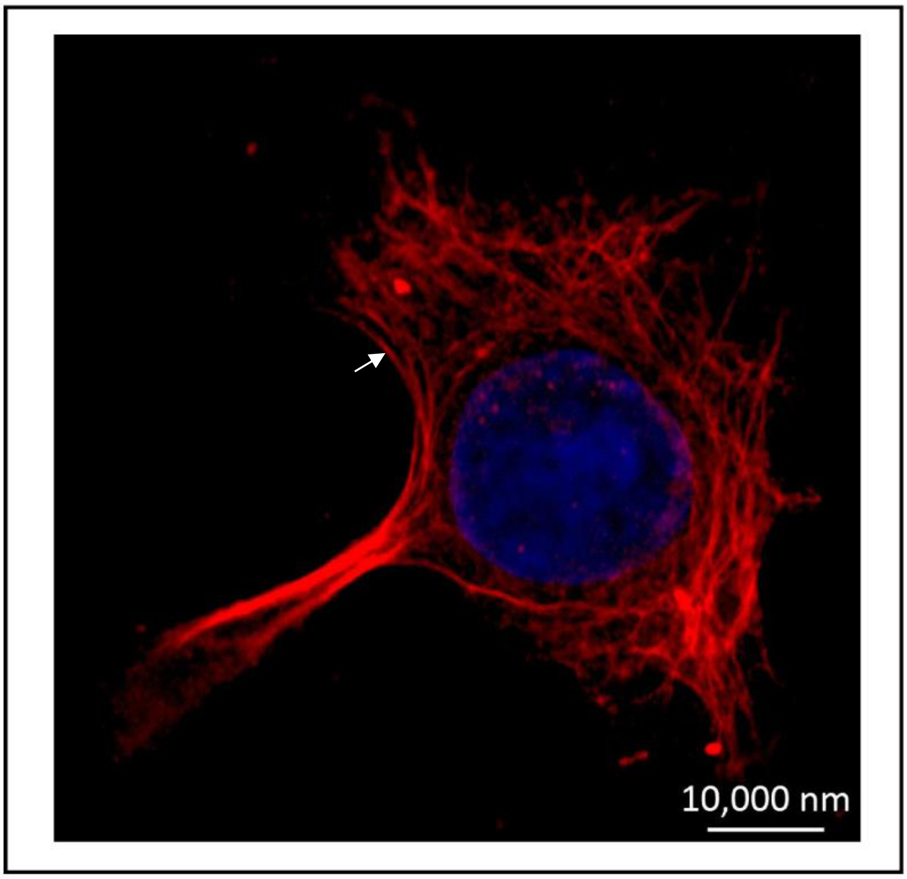

Supplementary Fig. 1. RABV M protein is clustered to form the filamentous network in the cytoplasm. N2a cells were infected by RABV at an MOI of 1 for 8 h. Subsequently, the cells were fixed, permeabilized, and incubated with the anti-RABV-M antibody (red). The nuclei (Nuc) were stained with DAPI (blue). The cells were analyzed using a laser scanning confocal microscopy. Scale bars, 10,000 nm. The white arrow represent the filamentous network of viral M protein.

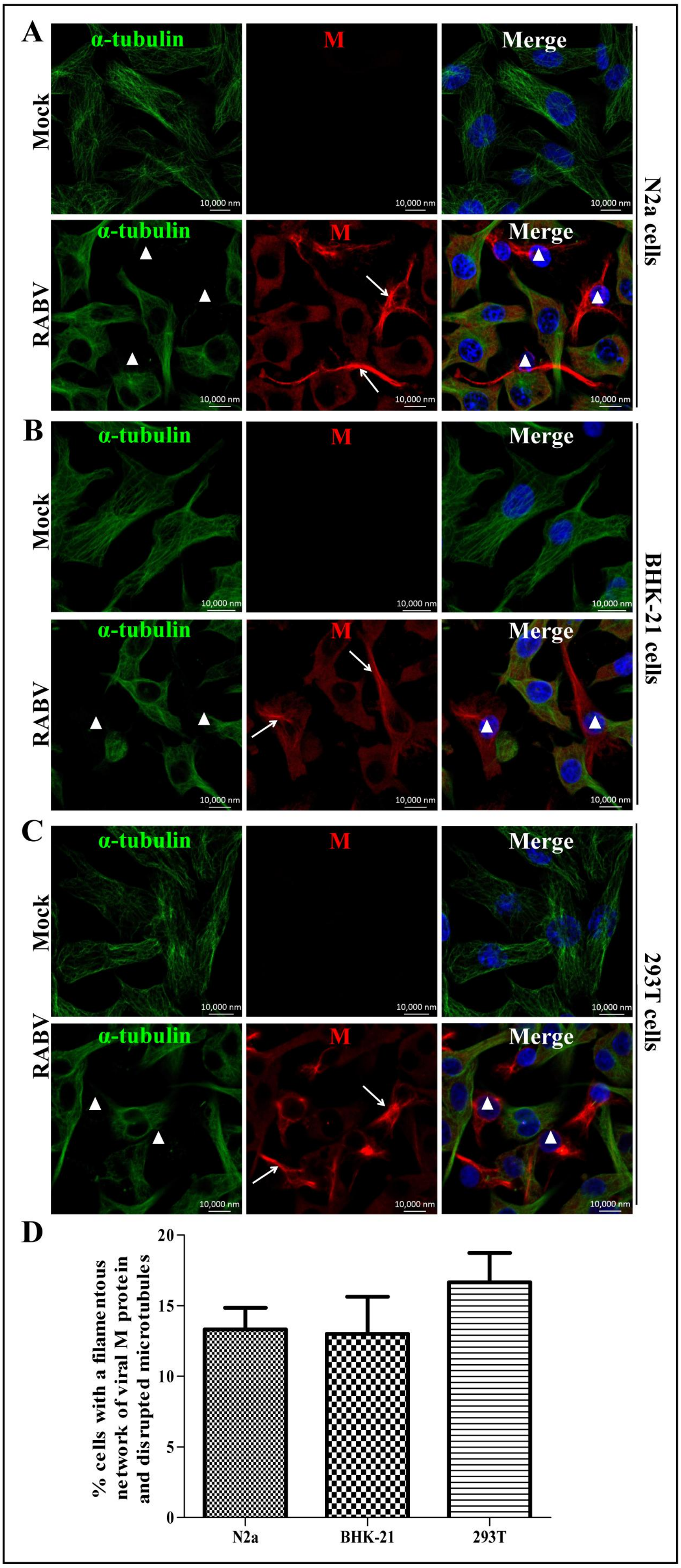
A
α-tubulin
M
Merge
Mock
RABV
N2a cells
B
BHK-21 cells
C
293T cells
10,000 nm
D
% cells with a filamentous network of viral M protein and disrupted microtubules
0
5
10
15
20
N2a
BHK-21
293T

Supplementary Fig. 2. RABV infection damages the host microtubule cytoskeleton. N2a cells (A), BHK-21 cells (B), and 293T cells (C) were either mock infected or infected by RABV at an MOI of 1 for 12 h. Subsequently, the cells were fixed, permeabilized, and incubated with the anti-α-tubulin antibody (green) and anti-RABV-M antibody (red). The nuclei (Nuc) were stained with DAPI (blue). The cells were analyzed using a laser scanning confocal microscopy. The white arrow represents the filamentous network of viral M protein in RABV-infected cells and the white triangle indicates the cell which has a filamentous network viral M protein and disrupted microtubules. Scale bars, 10,000 nm. (D) Three random microscopic fields having at least 50 cells each were chosen to calculate the number of cells with a filamentous network of viral M protein and disrupted microtubules at 12 hpi. Results were expressed as percentage of viral filamentous network-positive and cellular microtubules-negative cells relative to DAPI-positive cells. Data were represented as the means ± SD (n=3).

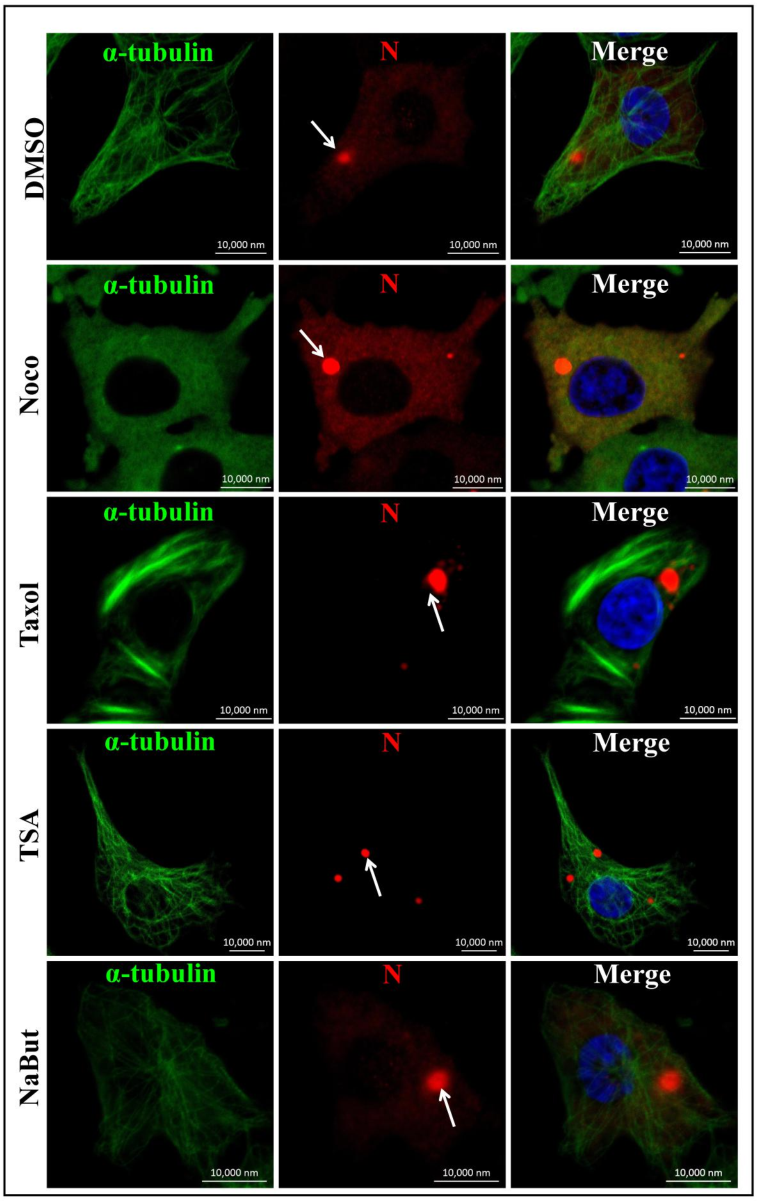

Supplementary Fig. 3. Effects of drugs treatment on the formation of NBs. N2a cells were infected with RABV for 4 h and then incubated with Noco, Taxol, TSA, NaBut or DMSO for another 20 h. The cells were fixed, permeabilized and then incubated with anti-α-tubulin antibody (green) and anti-RABV-N antibody (red). Nuclei (Nuc) were stained with DAPI (blue). The cells were analyzed using a laser scanning confocal microscopy. The white arrow indicates NBs in RABV-infected cells. Scale bars, 10,000 nm.

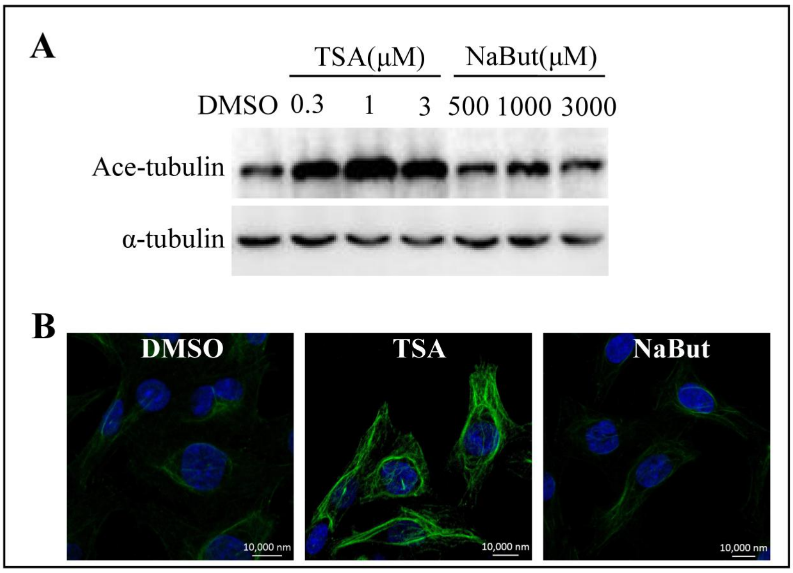

Supplementary Fig. 4. Effects of TSA or NaBut treatment on the acetylated modification of microtubules. (A) N2a cells were incubated with TSA, NaBut or DMSO (as control) for 20 h and then subjected to western blotting to determine the expressions of ace-tubulin and α-tubulin. (B) Ace-tubulin (green) in TSA (0.3 μM), NaBut (500 μM) or DMSO (500 μM)-treated N2a cells was detected by confocal microscopy. The cell nuclei (Nuc) were stained with DAPI (blue). Scale bars, 10,000 nm.
